# Ureteral cancer successfully treated with laparoscopic nephroureterectomy with temporary intraoperative occlusion of the internal iliac artery for pelvic arteriovenous malformation

**DOI:** 10.1002/iju5.12780

**Published:** 2024-09-06

**Authors:** Juntaro Koyama, Tomonori Sato, Yasufumi Sato, Shin Sato, Shuichi Shimada, Shinji Taniuchi, Ken Tsuchida, Masahiro Tsuboi, Kazuhiro Sakamoto, Yoshihiro Ikeda

**Affiliations:** ^1^ Department of Urology Osaki Citizen Hospital Osaki Japan; ^2^ Department of Urology Tohoku University Graduate School of Medicine Sendai Japan; ^3^ Department of Pathology Osaki Citizen Hospital Osaki Japan; ^4^ Department of Vascular Surgery Osaki Citizen Hospital Osaki Japan; ^5^ Department of Diagnostic Radiology Osaki Citizen Hospital Osaki Japan

**Keywords:** arteriovenous malformation, intraoperative internal iliac artery temporary occlusion, laparoscopic nephroureterectomy, ureteral cancer

## Abstract

**Introduction:**

Pelvic arteriovenous malformation is often a source of intraoperative bleeding. Here, we report our experience with a case of ureteral cancer with pelvic arteriovenous malformation treated using laparoscopic nephroureterectomy with temporary intraoperative occlusion of the internal iliac artery.

**Case presentation:**

A 75‐year‐old man presented to our hospital with asymptomatic macro‐hematuria. Contrast‐enhanced computed tomography revealed right ureteral tumor with no apparent metastases, and right pelvic arteriovenous malformation. Three months later, multiple bladder cancers were identified and the patient underwent trans‐urethral resection of bladder tumor. The pathological diagnosis was urothelial carcinoma, pTa. We performed right laparoscopic nephroureterectomy with temporary intraoperative occlusion of the internal iliac artery. There was little intraoperative bleeding, and the surgery was safely completed. The pathological diagnosis was urothelial carcinoma, pT2 + Tis.

**Conclusion:**

A patient showing ureteral cancer with pelvic arteriovenous malformation was safely treated using laparoscopic nephroureterectomy with temporary occlusion of the internal iliac artery.

**Abbreviations & Acronyms**
AVM: arteriovenous malformation
BCG: bacille Calmette‐Guérin
CT: computed tomography
TURBT: trans‐urethral resection of bladder tumor

**Keynote message**

We report a case of right ureteral cancer with pelvic arteriovenous malformation, which is often a source of intraoperative bleeding. Safe treatment was achieved by laparoscopic nephroureterectomy in which the internal iliac artery was temporarily occluded during surgery.

## Introduction

Pelvic AVM is relatively rare, but often represents a source of intraoperative bleeding during pelvic surgery.^1^, ^2^ Few reports have described the perioperative management of pelvic AVM in the field of urology. Here, we report a case of ureteral cancer with pelvic AVM treated using laparoscopic nephroureterectomy with temporary intraoperative occlusion of the internal iliac artery.

## Case presentation

**Fig. 1:**
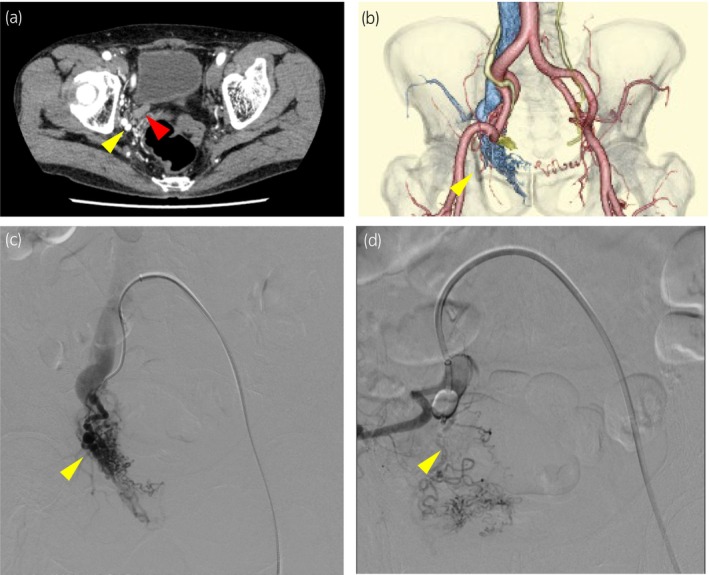
(a) and (b) Shows that enhanced CT reveals right ureteral tumor with right pelvic AVM near the right lower ureter. Arterial‐phase right internal iliac angiography shows numerous expanded arteries in the AVM (c). After inflating the occluding balloon, angiography confirms correct placement of the balloon in the right internal iliac artery and reveals stagnant flow (d). Red arrowhead shows the urethral tumor and yellow arrow shows the AVM.

**Fig. 2:**
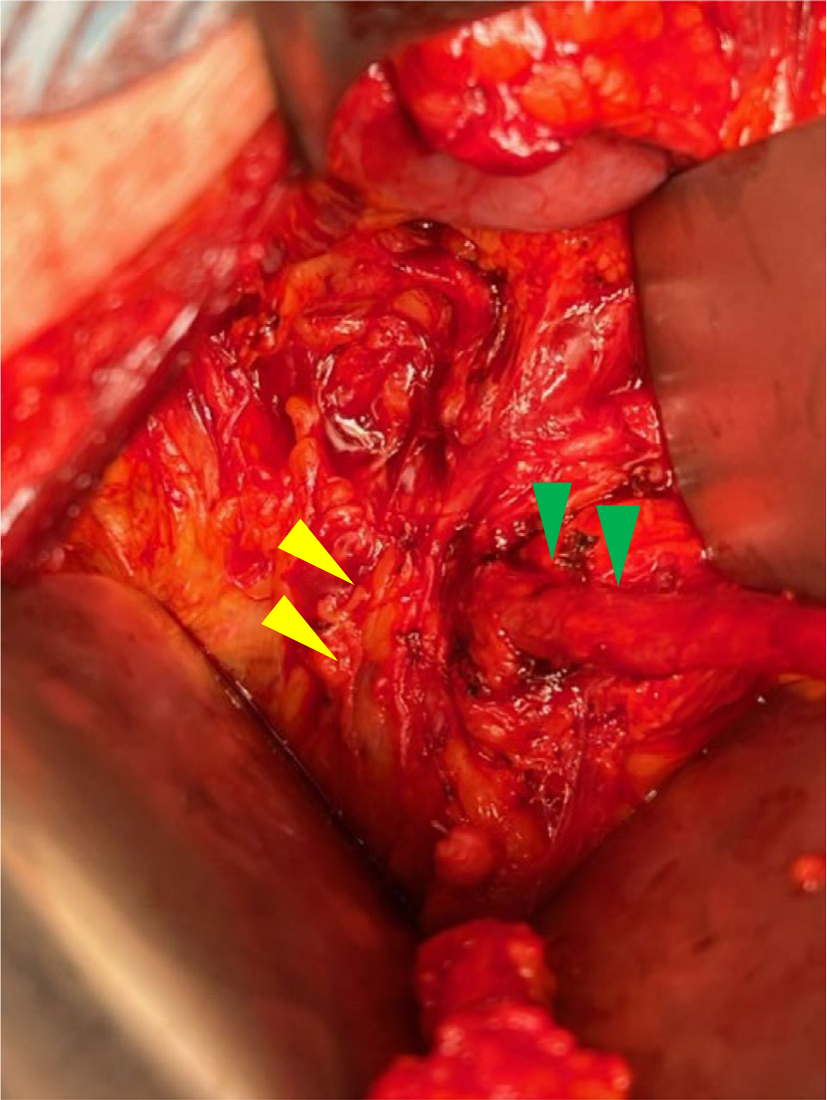
The pelvic AVM near the right ureteral tumor appears reduced in size while the internal iliac artery is occluded. Green arrowhead shows the ureter and yellow arrowhead shows the shrunken AVM.

A 75‐year‐old man presented to our hospital with asymptomatic macro‐hematuria. Contrast‐enhanced CT revealed right ureteral tumor with no metastatic lesions and right pelvic AVM near the right lower ureter (Fig. 1a,b). The patient hadn't complained of any symptoms attributable to AVM. Retrograde pyelography revealed a contrast defect lesion in the right lower ureter, but cytology showed no malignancy. No bladder tumor was identified. The patient requested follow‐up observations until malignant findings appeared. Three months later, multiple bladder cancers were identified and TURBT was performed. No change in the ureteral tumor was evident. The pathological diagnosis was urothelial carcinoma, pTa. We planned right laparoscopic nephroureterectomy. Preoperative CT showed a relatively extensive AVM in the arterial‐phase and hemostasis was considered likely to be difficult in the event of intraoperative vascular damage. CT also showed the ureter crossing the surface of the AVM. There was a risk of excessive bleeding during lower ureteral dissection, and vascular treatment would be required. The main vessel feeding the AVM was the right internal iliac artery, with apparent collateral circulation from the contralateral internal iliac artery. We referred the patient to the radiology department to plan prophylactic temporary balloon occlusion of the right internal iliac artery during the procedure. At the start of surgery, the patient was placed in a lateral decubitus position with the right side up and in a slight jack‐knife posture. A renal hilum procedure was performed using a retroperitoneal approach. The patient was then changed to a supine position. Pelvic angiography was performed using the left common femoral iliac artery. Arterial‐phase right internal iliac angiography showed numerous expanded arteries in the AVM and early drainage into the internal iliac vein (Fig. 1c). A 6‐Fr catheter with 13‐mm occlusive balloons (Selecon MP Catheter II; TERUMO JAPAN, Tokyo, Japan) was positioned with the tip in the proximal portion of the right internal iliac artery, just after the bifurcation from the common iliac artery.^3^ After inflating the occluding balloon, angiography confirmed correct placement of the balloon and revealed stagnant flow (Fig. 1d). Bladder cuff resection was then performed through a midline incision in the lower abdomen. With the temporary occlusion of the AVM, disappearance of the AVM in the surgical field was also confirmed (Fig. 2). During dissection of the lower ureter from the surrounding area, we were able to safely complete the procedure without any bleeding. Just before ending the surgery, we confirmed the absence of any bleeding and released the occlusion. After again confirming hemostasis, the incision in the abdomen was closed. Lymph node dissection was not performed. The ischemic time was 2 h 3 min. The volume of intraoperative bleeding was 10 mL. No adverse events associated with occlusion of the internal iliac artery, such as intermittent lameness or intestinal ischemia, were observed postoperatively. We were thus able to complete the surgery safely. The pathological diagnosis was urothelial carcinoma, pT2 + Tis. Adjuvant chemotherapy was not performed.

Three months later, the patient developed multiple recurrences of bladder cancer and underwent TURBT. The pathological diagnosis was urothelial carcinoma, pTa. We performed BCG intravesical injection therapy (80 mg, nine times). As of 6 months after the final TURBT, the patient has remained free from bladder recurrences and metastatic lesions.

## Discussion

To the best of our knowledge, this represents the first report of laparoscopic nephroureterectomy with temporary intraoperative occlusion of the internal iliac artery for pelvic AVM. Pelvic AVMs comprise arteries and veins connected through a central nidus that bypasses the high resistance provided by capillary beds.^4^ Such structures can cause symptoms ranging from transient discomfort to life‐threatening bleeding complications.^5^ Complications of untreated lesions or failure of treatment for pelvic AVMs include severe hemorrhagic hypovolemic shock, severe heart failure, and cauda equina and root compression leading to diplegia and neurogenic bladder.^6^, ^7^ The Schobinger classification system describes the clinical course of AVMs, from asymptomatic arteriovenous shunting (stage 1), through growth and functional symptoms, and finally to high‐output congestive heart failure and left ventricular hypertrophy (stage 4).^8^ In the present case, the AVM was stage 1 because of the absence of symptoms. Permanent treatment of the AVM was not considered necessary. According the ISSVA classification, AVMs are categorized as a simple vascular malformations just like arteriovenous fistulas (AVFs).^9^ No clear distinction between AVMs and AVFs has yet been made.

Intraoperative embolization of the internal iliac artery is a well‐studied treatment for placenta accreta spectrum.^10^, ^11^ Placenta accreta spectrum involves abnormal placental adhesion beyond the superficial myometrium, which may lead to severe life‐threatening hemorrhage requiring massive blood transfusions in the peripartum period. Prophylactic balloon catheterization of bilateral internal iliac arteries can prevent excessive intraoperative hemorrhage. However, few reports have discussed intraoperative arterial embolization in the field of urology, and urologists should be aware of the procedure. In the present case, the method of securing the internal iliac artery with a bulldog clamp during surgery was considered. However, if bleeding occurs during the surgery, we can perform endovascular therapy immediately when we choose to insert an occlusive balloon into the internal iliac artery. We therefore chose endovascular temporary occlusion.

In the present case, the internal iliac artery was occluded during the operation. Permanent intravascular embolization of AVM sometimes causes AVM recurrence via the collateral circulation.^4^ In such cases, anticipating the development of collateral vessels after permanent embolization is difficult, and hemostasis is expected to be challenging if vascular damage occurs. Further, catheter re‐embolization after permanent embolization is also difficult. In the present case, the AVM had developed collateral circulation from the contralateral internal iliac artery. We considered that the AVM was at high risk of recurrence after permanent embolization and opted for temporary intraoperative embolization. If bleeding occurred after the occlusion was released, we planned to provide treatment according to the severity of bleeding. In cases with minor bleeding, we could end the procedure with pressure hemostasis or the application of materials like Surgicel or TachoSil. In cases with major bleeding, we needed to add definitive treatment for the AVM through endovascular therapy. No complications such as thrombosis or dissection were observed in the present case, and intraoperative embolization was safely performed. This surgery must be performed in a hybrid operating room where intraoperative occlusion can be performed. Back‐up from a vascular surgeon is necessary as a precaution against intraoperative bleeding.

## Conclusion

We were able to safely perform laparoscopic nephroureterectomy with temporary intraoperative occlusion of the internal iliac artery in a case of ipsilateral pelvic AVM.

## Author contributions

Juntaro Koyama: Conceptualization; data curation; investigation; methodology; visualization; writing – original draft. Tomonori Sato: Supervision; validation. Yasufumi Sato: Validation. Shin Sato: Validation. Shuichi Shimada: Validation. Shinji Taniuchi: Validation. Ken Tsuchida: Methodology. Masahiro Tsuboi: Methodology. Kazuhiro Sakamoto: Validation. Yoshihiro Ikeda: Supervision; validation.

## Conflict of interest

The authors declare no conflict of interest.

## Approval of the research protocol by an Institutional Reviewer Board

Not applicable.

## Informed consent

Informed consent was obtained from the patient for publication of this case report and the accompanying images.

## Registry and the Registration No. of the study/trial

Not applicable.
